# Cul4a promotes zebrafish primitive erythropoiesis via upregulating *scl* and *gata1* expression

**DOI:** 10.1038/s41419-019-1629-7

**Published:** 2019-05-17

**Authors:** Fan Yang, Huili Hu, Yuanyuan Liu, Ming Shao, Changshun Shao, Yaoqin Gong

**Affiliations:** 1 The Key Laboratory of Experimental Teratology, Ministry of Education and Department of Genetics, School of Basic Medical Sciences, Shandong University, 250012 Jinan, China; 2 School of Life Sciences, Shandong University, 266237 Qingdao, China; 3 Institutes for Translational Medicine and National Key Laboratory of Radiation Medicine and Protection, Soochow University, 215123 Suzhou, China

**Keywords:** Embryogenesis, Erythropoiesis

## Abstract

CUL4A and CUL4B are closely related members in Cullin family and can each assemble a Cullin-RING E3 ligase complex (Cullin-RING Ligase 4A or 4B, CRL4A, or CRL4B) and participate in a variety of biological processes. Previously we showed that zebrafish *cul4a*, but not *cul4b*, is essential for cardiac and pectoral fin development. Here, we have identified *cul4a* as a crucial regulator of primitive erythropoiesis in zebrafish embryonic development. Depletion of *cul4a* resulted in a striking reduction of erythroid cells due to the inhibition of erythroid differentiation. Transcript levels for early hematopoietic regulatory genes including *scl*, *lmo2*, and *gata1* are significantly reduced in *cul4a*-deficient embryos. Mechanistically, we demonstrated that *scl* and *gata1*, the central regulators of primitive hematopoiesis for erythroid determination, are transcriptionally upregulated by *cul4a*. These findings demonstrate an important role for *cul4a* in primitive erythropoiesis and may bear implications in regeneration medicine of anemia and related diseases.

## Introduction

Zebrafish (*Danio rerio*) has become a powerful model for investigating hematopoiesis because its transparency greatly facilitates the visualization of hematopoietic system. The Zebrafish hematopoietic system is highly analogous to that in mammals, including the phenotype of blood cells and the key hematopoietic-lineage genes^1,2^. Zebrafish hematopoiesis consists of two successive waves, primitive and definitive hematopoiesis^3^. The first wave generates primitive erythrocytes (primitive erythropoiesis) and macrophages (primitive myelopoiesis), and the second wave produces hematopoietic stem cells (HSCs)^4^. The primitive myelopoiesis occurs at the anterior lateral plate mesoderm, while primitive erythropoiesis takes place at the posterior lateral plate mesoderm (PLPM), which subsequently forms intermediate cell mass (ICM) at the midline^5,6^. A sophisticated network of many transcription factors has been described to regulate primitive and definitive hematopoiesis^7^. Among them, the stem cell leukemia gene (Scl/Tal1) is a central regulator of primitive hematopoiesis^8,9^. Gata1, a zinc finger protein, is specifically required for the maturation of proerythroblasts, and Pu.1, a transcription factor that contains an ETS domain, plays an indispensable role in primitive myelopoiesis^10,11^. In contrast, definitive hematopoiesis is initiated by the transcription factors of Runx1 and c-Myb^6^. Although the importance of these transcription factors has been demonstrated in cell-based ex vivo assays as well as in knockout mouse models, the regulation of their expression remains poorly understood.

Cullin-RING E3 ligase (CRL) complexes, which contain Cullin, RING protein, and substrate-recognition subunit as the core components, represent the largest known class of ubiquitin ligases and participate in a broad variety of physiologically and developmentally controlled processes^12,13^. Among Cullin family members, CUL4A and CUL4B have the highest degree of homology, with ~80% identity in protein sequences, and are believed to be derived from one common ancestor CUL4^14–16^. There is only one ortholog, *cul4*, in lower organisms. As the scaffold protein, CUL4 assembles with damaged DNA-binding protein 1 at its N terminus and with ROC1 at its C terminus to form CRL4 complexes that target different substrates for proteosomal degradation or for protein modification^17,18^. Although CUL4A and CUL4B share a high degree of homology, studies have shown that the two *CUL4* genes in mammals are not entirely redundant. Loss of function mutations in human *CUL4B* cause mental retardation, short stature, abnormal gait, impaired speech, and other abnormalities^19–21^. *Cul4b* null mice are embryonic lethal and tissue-specific *Cul4b* knockout mice exhibited a variety of developmental defects^22–26^. In contrast, no germline mutations in human *CUL4A* gene have been reported, and *Cul4a* knockout mice exhibited no apparent developmental phenotype except that the knockout males were sterile^27,28^. As in mammals, there are two *cul4* members, *cul4a* and *cul4b*, in zebrafish. The two genes have a high degree of homology with their respective mammalian counterparts *CUL4A* and *CUL4B*^29^. We previously reported that *cul4a*, but not *cul4b*, is essential for zebrafish cardiac development and pectoral fin formation^29^. These findings indicate that the functions of cul4 paralogs are varied in different species. In this study, we determined the expression profile of *cul4a* during zebrafish embryogenesis and found it was localized in primary hematopoietic region. Using morpholino and recently developed four-guide Cas9 RNP targeting technology^30^, we demonstrated that primitive erythropoiesis was significantly reduced after *cul4a* ablation. Mechanistically, Cul4a was shown to be required for the expression of *scl* and *gata1* via promoting H3K4me3. Our results revealed a crucial role for Cul4a in zebrafish primitive hematopoiesis.

## Results

### Knockdown of *cul4a* in zebrafish significantly reduces the number of erythrocytes

To assess whether cul4a is involved in the blood cell development in zebrafish, we firstly examined the expression pattern of *cul4a* during zebrafish embryonic development using whole-mount in situ hybridization (WISH). Maternally supplied *cul4a* transcripts were distributed evenly in all blastomeres through the blastula and gastrulation stages (Fig. S1a, b). Then, it was found to express elusively in two primitive hematopoietic sites, the PLPM at 12 h post fertilization (hpf) (Fig. S1c, d) and in the ICM at 24 hpf (Fig. S1e). After 48 hpf, *cul4a* was hardly detectable in caudal hematopoietic tissue where definitive hematopoiesis occurs (Fig. S1f). This spatiotemporal distribution pattern of *cul4a* expression corresponds to that of primitive hematopoiesis.

**Fig. 1:**
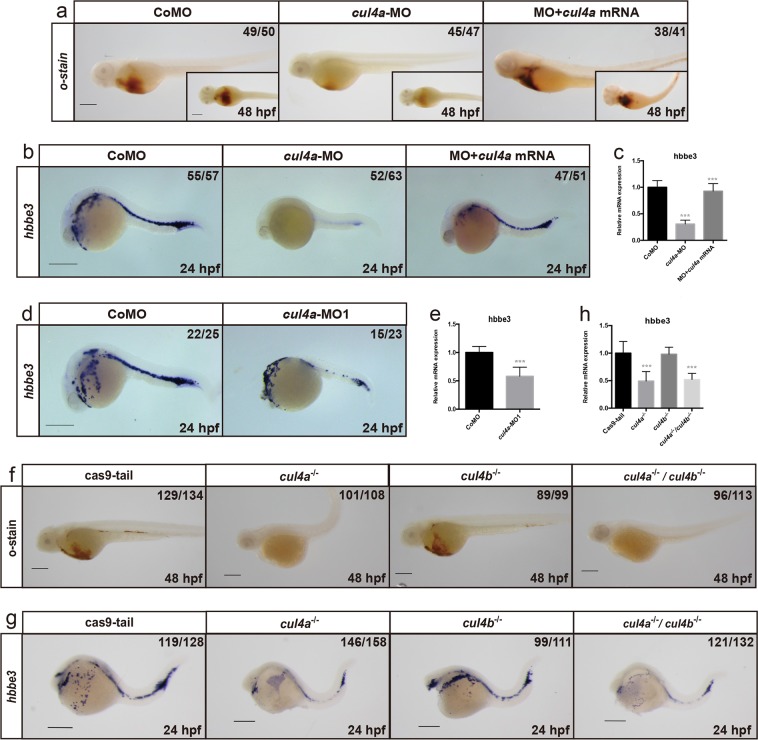
Knockdown of *cul4a* in zebrafish significantly reduces the number of erythrocytes. **a** *o*-dianisidine staining showed depletion of erythrocytes in *cul4a*-morphant embryos, compared with controls. Zebrafish *cul4a* mRNA rescued primitive erythropoiesis in *cul4a*-morphant embryos. **b** Expression of the embryonic hemoglobin, *hbbe3*, was analyzed by WISH at 24 hpf in embryos injected with CoMO or *cul4a*-MO, or MO coinjected with zebrafish *cul4a* mRNA. Lateral views are shown with anterior to the left. **c** Relative mRNA level of *hbbe3* was assayed by qRT-PCR in embryos injected with CoMO or *cul4a*-MO, or MO coinjected with *cul4a* mRNA at 24 hpf. **d** WISH was performed with *hbbe3* probes in embryos at 24 hpf injected with CoMO or *cul4a*-MO1. **e** Relative mRNA level of *hbbe3* was measured by qRT-PCR in embryos injected with CoMO or *cul4a*-MO1. **f** *o*-dianisidine staining showed decreased erythrocytes in *cul4a*^*−/−*^ and double knockout, but not in *cul4b*^*−/−*^ knockout embryos. Embryos shown are lateral views with anterior to the left. **g** The images of WISH with *hbbe3* mRNA probes in control (cas9-tail injected), *cul4a* ^−/−^, *cul4b* ^−/−^, and double knockout mutants at 24 hpf. **h** Relative mRNA level of *hbbe3* was assayed by qRT-PCR in control (cas9-tail injected), *cul4a*^−/−^, *cul4b*^−/−^, and double knockout mutants at 24 hpf. The number in the top right-hand corner indicates the phenotypic embryos/total embryos. qRT-PCR experiments were performed in triplicate. ***p < 0.001. All scale bars represent 250 μm

Our previous study showed that embryos injected with *cul4a*-MO (a splicing blocking morpholino) or *cul4a*-MO1 (a translational-blocking morpholino) exhibited an array of morphological defects, including tail curling, hindbrain edema, stunted or completely absent pectoral fins and pericardial edema that was accompanied by heart looping impairment^29^. Moreover, blood cells were markedly reduced or absent in the circulation of *cul4a* morphants. We therefore here focused on the hematopoiesis in the embryos injected with *cul4a*-MO, or *cul4a*-MO1 or control MO (CoMO) as described in our previous study^29^. Indeed, the *cul4a* morphants had significantly decreased number of red blood cells as shown by *o*-dianisidine staining (Fig. 1a). To rule out that the reduction in erythrocytes in *cul4a* morphants was due to heart defects, we performed WISH and quantitative real-time PCR (qRT-PCR) assays for globin transcripts in *cul4a* morphants at 24 hpf, when heart tubes in *cul4a* morphants were indistinguishable from those in controls. As shown in Fig. 1b, c, the expression of *hbbe3* was significantly downregulated in *cul4a* morphants compared to controls. To determine whether the hematopoietic defects observed in *cul4a* morphants resulted from a nonspecific morpholino effect, we performed rescue experiments using a mismatch *cul4a* mRNA to avoid targeting by cul4a-MO. After coinjection of *cul4a-*MO together with *cul4a* mRNA, the decreased erythrocytes in *cul4a* morphants could be efficiently rescued (Fig. 1a–c). Furthermore, embryos injected with *cul4a*-MO1 exhibited similar defects compared with the control embryos (Fig. 1d, e). To further confirm these phenotypes, we generate *cul4a*^*−/−*^, *cul4b*^*−/−*^, or double knockout mutants using an optimized four-guide Cas9 RNP targeting system^30^. Sequencing analysis showed that four-guide sgRNAs (a, b, d, and f for *cul4a*, a′, b′, d′, and e′ for *cul4b* in Table S1) could efficiently delete *cul4a* and *cul4b* gene, respectively (Fig. S1g, h). As shown in Fig. 1f–h, depletion of *cul4a*, but not *cul4b*, resulted in a significant reduction in the number of erythrocytes as demonstrated by *o*-dianisidine staining or *hbbe3* expression. Moreover, double knockout mutants exhibited similar phenotypes to *cul4a* knockout mutants. These results indicated that *cul4a* is required for primitive hematopoiesis.

### Zebrafish *cul4a* regulates the primitive erythropoiesis, but not primitive myelopoiesis or definitive hematopoiesis

**Fig. 2:**
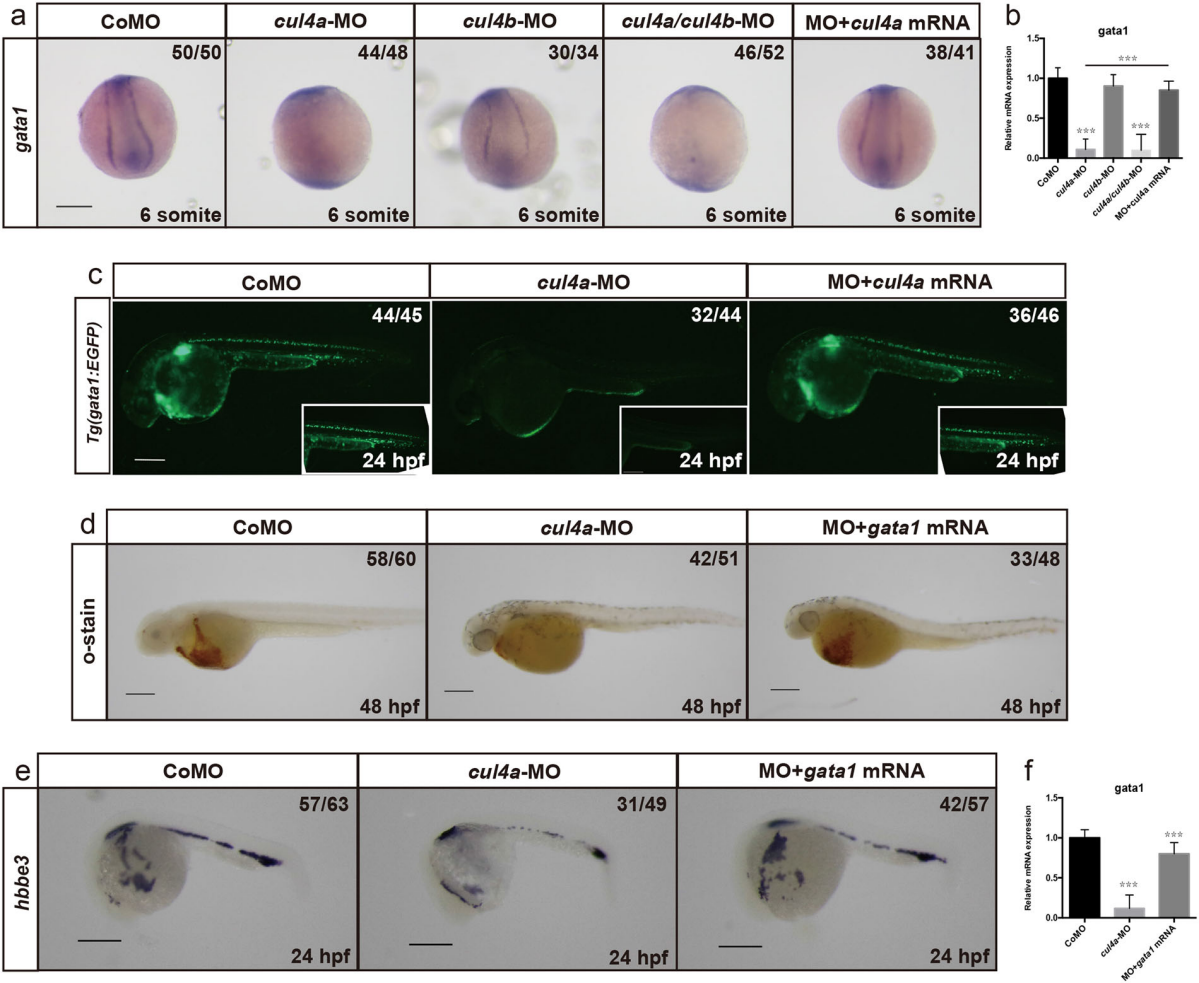
Zebrafish *cul4a* regulates the primitive erythropoiesis. **a** *Gata1* was analyzed by WISH in embryos at 6 somite injected with CoMO, *cul4a*-MO, *cul4b*-MO, *cul4a/cul4b*-MO, or *cul4a*-MO coinjected with zebrafish *cul4a* mRNA. **b** Relative *gata1* expression was analyzed by qRT-PCR in embryos injected with CoMO, *cul4a*-MO, *cul4b*-MO, *cul4a/cul4b*-MO, or MO coinjected with *cul4a* mRNA, respectively. **c** *cul4a*-MO was injected into *Tg (gata1: EGFP)* transgenic embryos and decreased EGFP-positive cells were observed. Coinjection of *cul4a* mRNA rescued the reduction in *gata1*^+^ cells caused by *cul4a* MOs. **d**–**f** Effects of *gata1* mRNA in rescuing hematopoietic defects in *cul4a*-morphant embryos as demonstrated by staining of *o*-dianisidine (**d**), WISH (**e**) and qRT-PCR (**f**) of *hbbe3* probes. The number in the top right-hand corner indicates the phenotypic embryos/total embryos. qRT-PCR experiments were performed in triplicate. ***p < 0.001. All scale bars represent 250 μm

Hematopoiesis occurs in two successive waves and is regulated by lineage-specific genes. *Gata1* and *pu.1* encode two transcription factors that regulate primitive erythropoiesis and primitive myelopoiesis, respectively^3,37,38^. To assess the role of *cul4a* in primitive hematopoiesis, we performed WISH in *cul4a* morphants for *gata1*, a marker for erythroid progenitors. Consistent with the scarcity of erythrocytes in *cul4a* morphants, the expression level of *gata1* was dramatically downregulated by *cul4a* knockdown (Fig. 2a). Coinjection of in vitro synthesized *cul4a* mRNA rescued the decreased expression of *gata1* in *cul4a* morphants (Fig. 2a). qRT-PCR assays further confirmed the reduction in *gata1* expression in *cul4a* morphants and its rescue by *cul4a* mRNA (Fig. 2b). Consistent with unaltered number of erythrocytes in *cul4b*-deficient embryos, *gata1* expression was not affected by *cul4b* knockdown (Fig. 2a, b). To further confirm this finding, we injected *cul4a*-CoMO or *cul4a*-MO into zebrafish embryos of the transgenic line *Tg (gata1: EGFP)* in which the differentiated erythroid cells are labeled by EGFP. We found that the EGFP-positive population was substantially reduced in *cul4a* morphants at 24 hpf, and the reduction in EGFP-positive cells was efficiently reversed by coinjection of *cul4a* mRNA (Fig. 2c). To determine whether decreased number of erythrocytes was mediated by downregulation of *gata1* expression, we performed rescue experiments using in vitro synthesized *gata1* mRNA. As shown in Fig. 2d–f, coinjection of in vitro synthesized *gata1* mRNA could efficiently block the reduction in the number of erythrocytes caused by *cul4a* depletion. These results indicated that *cul4a* promotes primitive erythropoiesis by regulating *gata1* expression.

**Fig. 3:**
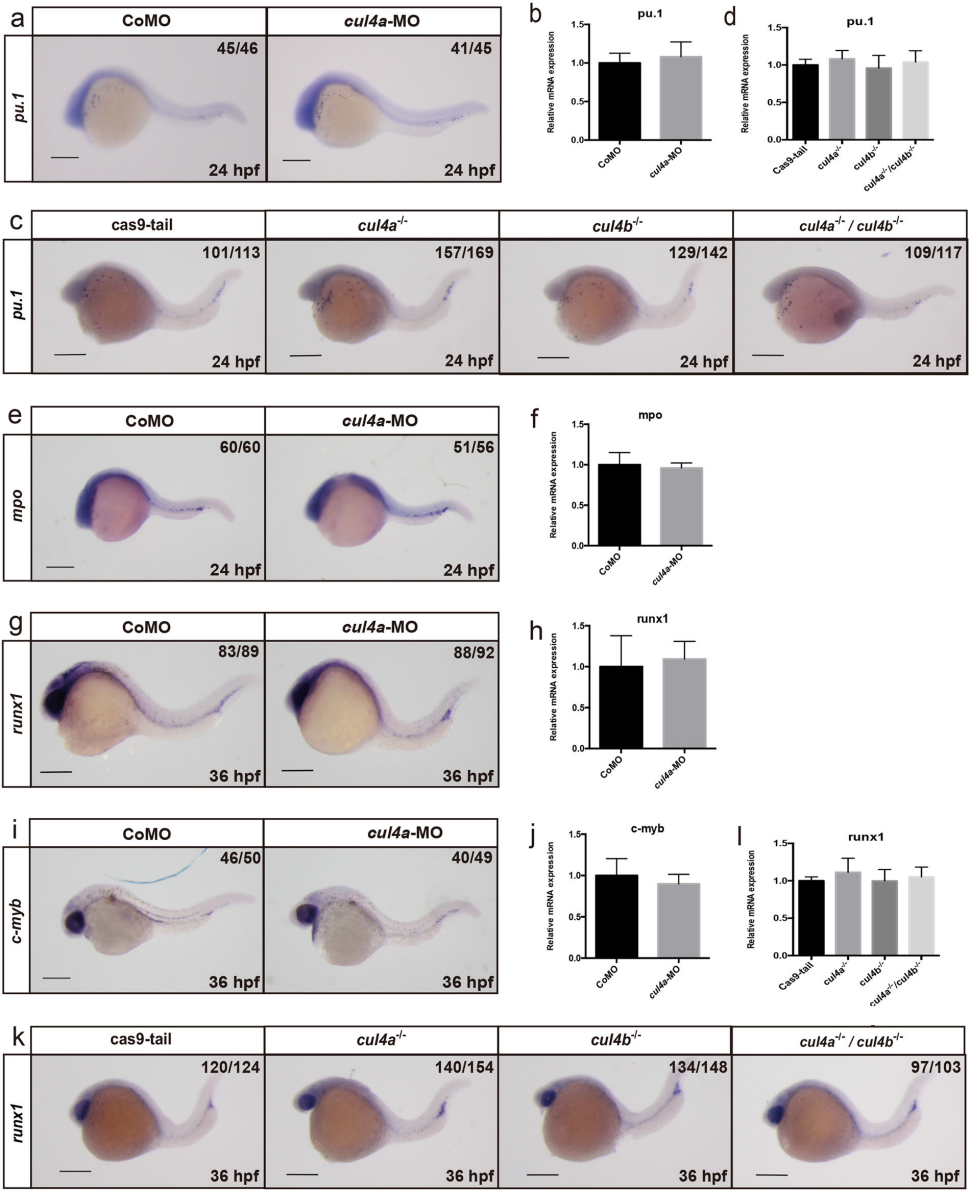
Downregulation of zebrafish *cul4a* has no effect on primitive myelopoiesis or definitive hematopoiesis. **a** *Pu.1* was analyzed by WISH at 24 hpf in embryos injected with CoMO or *cul4a*-MO. **b** Relative expression of *pu.1* mRNA was analyzed by qRT-PCR in embryos injected with CoMO or *cul4a*-MO. **c**, **d** Expression of *pu.1* in control and *cul4a*^*−/−*^, *cul4b*^*−/−*^, and double knockout mutants was assayed by WISH (**c**) and by qRT-PCR (**d**). **e**, **f** *Mpo* was analyzed by WISH (**e**) and qRT-PCR (**f**) at 24 hpf in embryos injected with CoMO or *cul4a*-MO. **g**–**j** *runx1* and *c-myb* expression were analyzed by WISH (**g**, **i**) or qRT-PCR (**h**, **j**) in embryos injected with CoMO or *cul4a*-MO. **k**, **l** Expression of *runx1* in control and *cul4a*^*−/−*^, *cul4b* ^*−/−*^, and double knockout embryos. The number in the top right-hand corner indicates the phenotypic embryos/total embryos. qRT-PCR experiments were performed in triplicate. All scale bars represent 250 μm

We then examined the role of *cul4a* in the development of myeloid cells during primitive hematopoiesis. The expression levels of *pu.1*, a marker for myeloid progenitors, were comparable between *cul4a* morphants and CoMO-injected embryos, as indicated by WISH and qRT-PCR (Fig. 3a, b). To confirm these results, we examined the *pu.1* expression in *cul4a*^−/−^, *cul4b*^−/−^, and double knockout mutants. As shown in Fig. 3c, d, depletion of *cul4a* or *cul4b* or both did not alter the expression of *pu.1*. Similarly, the expression of *mpo*, a marker for granulocytes, was not affected by *cul4a* MOs (Fig. 3e, f). These results suggest that both *cul4a* and *cul4b* are dispensable for the development of myeloid cells during primitive hematopoiesis.

We also assessed whether Cul4a affected definitive hematopoiesis. WISH and qRT-PCR were performed with markers for definitive hematopoiesis, *runx1* and *c-myb*. The expression of neither *runx1* nor *c-myb* in *cul4a* morphants appeared to be affected by *cul4a* morpholinos (Fig. 3g–j). Similarly, the expression of *runx1* was not affected by depletion of *cul4a*, *cul4b*, or both *cul4a* and *cul4b* (Fig. 3k, l). These data indicated that lack of *cul4a* and *cul4b* has no impact on the emergence of primitive myeloid progenitors or definitive stem cells.

### The expression of the early hematopoietic markers *scl* and *lmo2* is downregulated in *cul4a* morphants

**Fig. 4:**
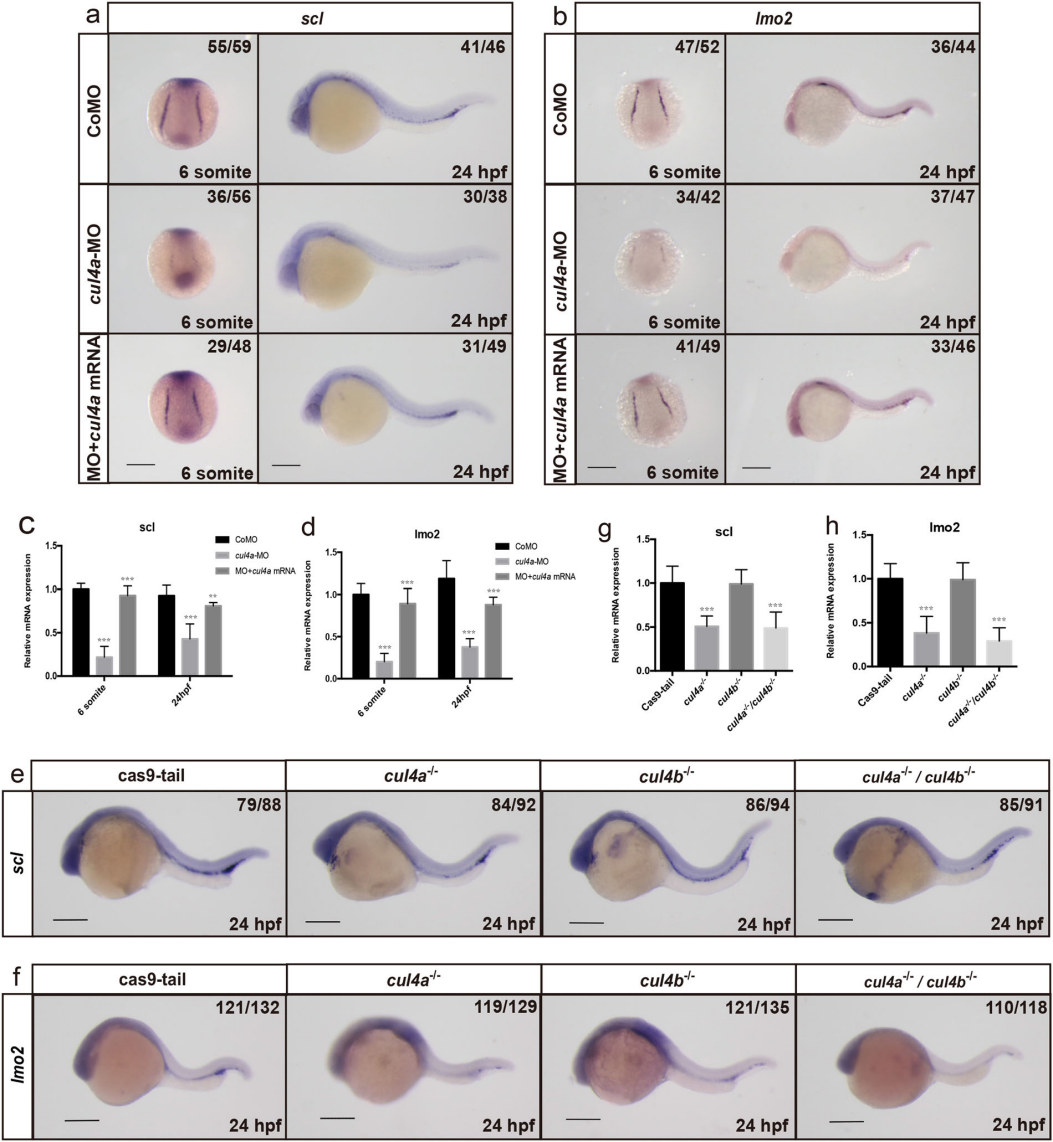
The expression of the early hematopoietic markers *scl* and *lmo2* was downregulated in *cul4a* mutants. **a**, **b** WISH was performed with *scl* (**a**) or *lmo2* (**b**) probes in embryos at 6 somite and 24 hpf, respectively. Embryos at 6-somite stage were in poster order view with anterior to the top. The ICM regions of embryos were lateral views with anterior to the left. **c**, **d** qRT-PCR analyzed the expression of *scl* and *lmo2* in embryos injected with CoMO or *cul4a*-MO, or MO coinjected with zebrafish *cul4a* mRNA at 6 somite and 24 hpf. **e**, **f** The images of WISH with *scl* or *lmo2* mRNA probes in control (cas9-tail injected), *cul4a* ^*−/−*^, *cul4b* ^*−/−*^, and double knockout mutants at 24 hpf. **g**, **h** Relative expression of *scl* and *lmo2* mRNA were analyzed by qRT-PCR in control and *cul4a* ^*−/−*^, *cul4b* ^*−/−*^, and double knockout mutants. The number in the top right-hand corner indicates the phenotypic embryos/total embryos. qRT-PCR experiments were performed in triplicate. ***p < 0.001. All scale bars represent 250 μm

To investigate the molecular mechanism by which Cul4a regulates primitive erythropoiesis, we examined the expression of early hematopoietic progenitor markers *scl* and *lmo2*, which are required for the generation of hematopoietic progenitors within PLPM. The expression levels of these two genes were remarkably reduced in *cul4a*-MO-injected embryos compared with those in embryos injected with CoMO (Fig. 4a, b). The downregulation of *scl* and *lmo2* in *cul4a* morphants was confirmed by qRT-PCR assays (Fig. 4c, d). Importantly, coinjection of *cul4a* mRNA significantly blocked the reduction in the expression levels of *scl* and *lmo2*, both confirmed by WISH and qRT-PCR (Fig. 4a–d). We further confirmed that *scl* and *lmo2* expression levels were uniquely decreased in *cul4a*^*−/−*^ mutants rather than *cul4b*^*−/−*^ or double knockout mutants (Fig. 4e–h).

### Impaired primitive erythropoiesis caused by lack of *cul4a* is mediated by downregulated expression of *scl-α*

**Fig. 5:**
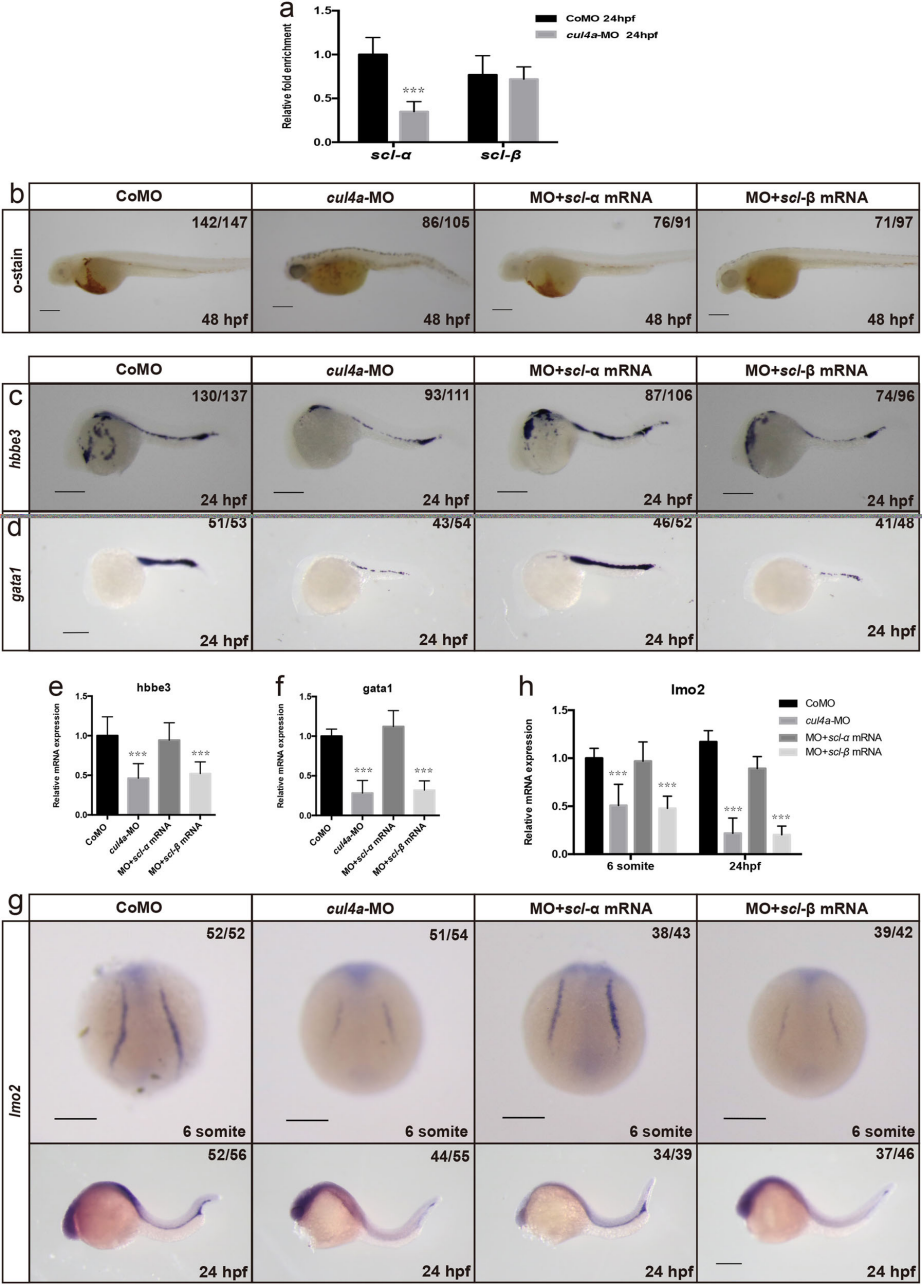
Impaired primitive erythropoiesis caused by *cul4a* knockdown is mediated by downregulated expression of *scl*. **a** The relative expression of the two isoforms of *scl*, *scl-α* and *scl-β*, were measured by qRT-PCR in embryos injected with CoMO or *cul4a*-MO. **b** *o*-dianisidine staining showed depletion of erythroid cells in *cul4a*-morphant embryos compared with controls. Zebrafish *scl-α*, but not *scl-β*, mRNA rescued the reduction in primitive erythropoiesis of *cul4a*-morphant embryos. **c**, **d** WISH was performed with *gata1* or *hbbe3* probes in 24 hpf embryos injected with CoMO or *cul4a*-MO, or MO coinjected with zebrafish *scl-α* or *scl-β* mRNA. **e**, **f** Relative expression of *gata1* or *hbbe3* in embryos injected with CoMO or *cul4a*-MO, or MO coinjected with zebrafish *scl-α* or *scl-β* mRNA at 24 hpf. **g** WISH was performed with *lmo2* probes in embryos at 6 somite and 24 hpf, respectively. Reduced expression of *lmo2* in *cul4a*-morphant embryos were rescued by coinjection of zebrafish *scl-α*, but not *scl-β* mRNA. **h** Relative expression of *lmo2* was measured by qRT-PCR in embryos injected with CoMO or *cul4a*-MO, or MO coinjected with zebrafish *scl-α* or *scl-β* mRNA at 6 somite and 24 hpf. The number in the top right-hand corner indicates the phenotypic embryos/total embryos. qRT-PCR experiments were performed in triplicate. ***p < 0.001. All scale bars represent 250 μm

It has been shown that zebrafish produces two *scl* isoforms, full-length *scl-α* and a shorter isoform *scl-β*, through alternative promoter sites. While *scl-α* and *scl–β* are redundant for the initiation of primitive hematopoiesis, *scl-β*, but not *scl-α*, is required for specification of definitive HSCs^39^. The fact that lack of *cul4a* impaired primitive erythropoiesis, but not definitive hematopoiesis, suggests that Cul4a regulates the expression of *scl-α*, but not that of *scl-β*. To confirm this notion, we used specific primer pairs to distinguish two isoforms. As expected, knockdown of *cul4a* in zebrafish resulted in a downregulation of *scl-α*, but had no effect on the expression of *scl-β* (Fig. 5a). To determine whether the impaired erythropoiesis caused by *cul4a* depletion is also medicated by the downregulation of *scl*, we performed rescue experiments with in vitro synthesized *scl* mRNA. We found that coinjection of in vitro synthesized *scl-α* mRNA rather than *scl-β* rescued the decreased primitive erythropoiesis in *cul4a* morphants (Fig. 5b). Consistently, the reduced expression of lineage-specific genes *gata1* and *hbbe3* was rescued by coinjection with *scl-α* mRNA as indicated by WISH and qRT-PCR (Fig. 5c–f). Interestingly, coinjection of *scl-α* mRNA also blocked the reduction in the expression of *lmo2* (Fig. 5g, h), suggesting that the reduction of *lmo2* is caused by the downregulation of *scl-α*. Taken together, these observations indicate that the impaired erythropoiesis due to *cul4a* knockdown is mediated by the downregulation of *scl-α* expression.

### *Cul4a* activates *scl-α* and *gata1* transcription by an epigenetic mechanism

**Fig. 6:**
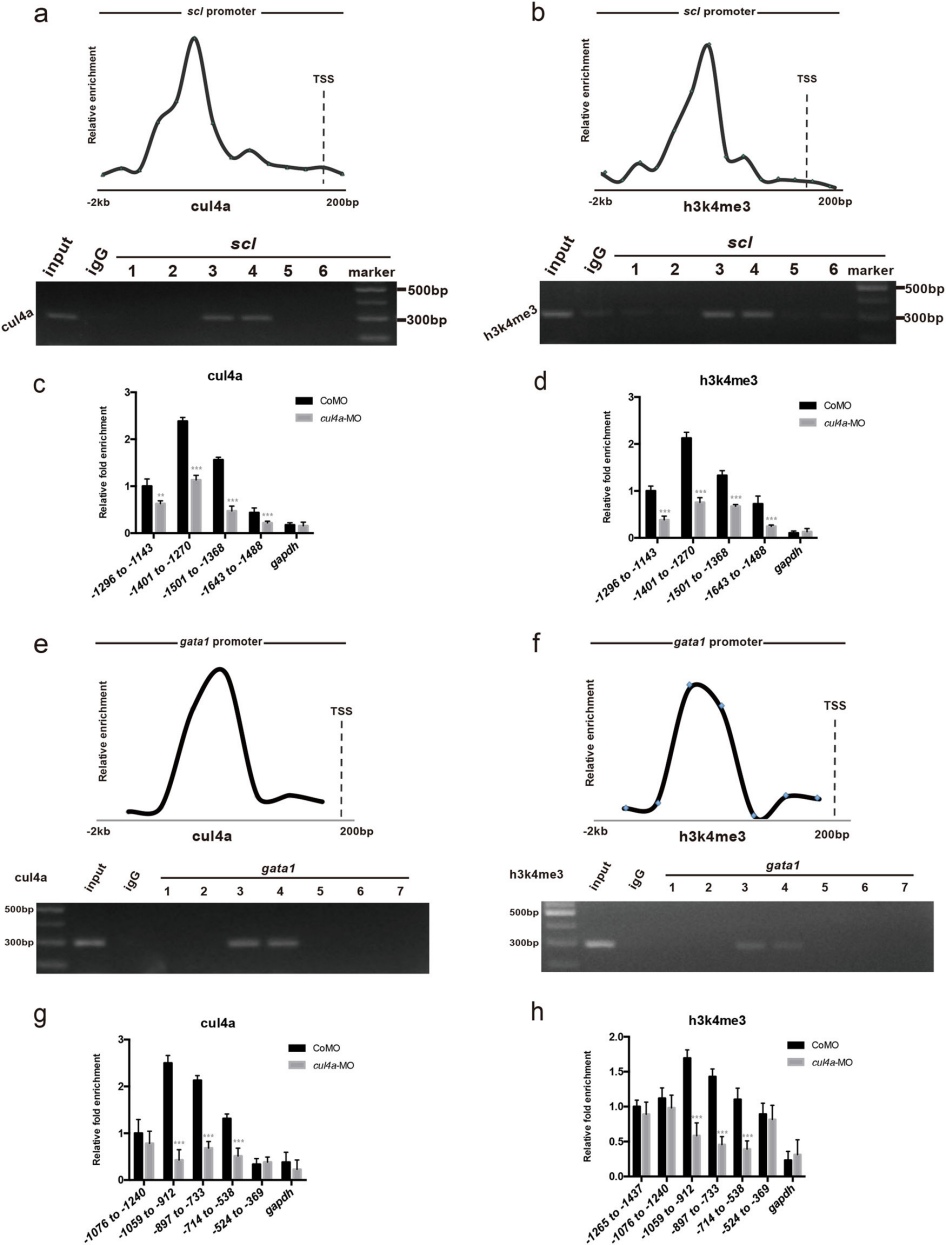
*Cul4a* activates *scl-α* and *gata1* transcription by an epigenetic mechanism. **a**, **b** E-qChIP assays of wild-type zebrafish at 24 hpf with antibodies against Cul4a (**a**) and H3K4me3 (**b**). The presence of *scl* promoter sequences in the input DNA and antibody-bound chromatin segments was analyzed by qPCR and agarose gel electrophoresis. **c**, **d** E-qChIP assays of *scl* at 24 hpf embryos injected with CoMO or *cul4a*-MO. **e**, **f** The presence of *gata1* promoter sequences in the input DNA and antibody-bound chromatin segments was analyzed by qPCR and agarose gel electrophoresis. **g**, **h** E-qChIP assays of *gata1* at 24 hpf embryos injected with CoMO or *cul4a*-MO. Experiments were performed in triplicate. ***p < 0.001, **p < 0.01

To further characterize whether *cul4a* regulates *scl-α* expression directly by binding to its promoter, we next performed a whole-embryo quantitative chromatin immunoprecipitation (E-qChIP) analysis to test whether Cul4a binds to *scl-α* promoter using six pairs of primers covering a region approximately ranging from −2000 bp to +200 bp of *scl-α* transcription start site. The E-qChIP assay revealed that the Cul4a occupancy peaked in the region at around −1643 bp to −1143 bp of the *scl* promoter (Fig. 6a). Notably, H3K4me3, a histone marker for transcription activation, was also enriched in the same region (Fig. 6b). We next examined the effect of *cul4a* knockdown on the enrichment of these proteins on the *scl–α* promoter and found that accompanying a marked reduction in the enrichment of *cul4a* bound to the *scl* promoter, the level of H3K4me3 at the *scl* promoter was also markedly decreased when *cul4a* was knocked-down (Fig. 6c, d). These results show that Cul4a can bind to *scl-α* promoter and thereafter promotes H3K4 trimethylation en route to activate *scl-α* transcription.

The facts that depletion of *cul4a* downregulated the expression of *gata1* and coinjection of in vitro synthesized *gata1* mRNA could efficiently block the reduction of erythrocytes in *cul4a* morphants suggests that Cul4a might directly regulate the transcription of *gata1* gene. To test this hypothesis, we used the ChIP assay to examine whether *gata1* gene is bound by Cul4a using seven pairs of primers specific for a region located approximately −2000 bp to +200 bp of *gata1* transcription start site. The ChIP assay showed that Cul4a could directly bind to the region at −1059 bp to −538 bp of *gata1* promoter. H3K4me3 was also bound to the same region. Moreover, the quantitative ChIP assay showed that knockdown of *cul4a* significantly decreased the binding of cul4a on the *gata1* promoter (Fig. 6g). Consistently, the level of H3K4me3 at the *gata1* promoter was also markedly decreased when *cul4a* was depleted (Fig. 6h). Together, these data show that *cul4a* can activate *gata1* transcription by promoting H3K4 trimethylation.

## Discussion

Many transcription factors regulating primitive and definitive hematopoiesis have been identified and mechanistically characterized. However, the regulation of their expression is less understood. In this study we showed that Cul4a is essential for zebrafish primitive erythropoiesis. Depletion of *cul4a* resulted in a severe reduction of the number of erythrocytes. We obtained several lines of evidences that support the conclusion that zebrafish Cul4a is directly involved in embryonic erythrocyte development. First, the reduction in the number of red blood cells was observed as early as 24 hpf, when heart tubes in *cul4a* morphants were indistinguishable from those in controls. Second, depletion of *cul4a* results in the downregulation of *gata1*, a gene essential of primitive erythroid-lineage development, which was efficiently restored by coinjection of *cul4a* mRNA. Third, knockdown of *cul4a* leads to significant downregulation of *scl*, a key transcription factor in primitive hematopoiesis. Furthermore, coinjection of zebrafish *scl-α* mRNA is able to restore erythropoiesis as well as *gata1* expression. Finally, the ChIP assay shows that Cul4a directly binds to both *scl-α* and *gata1* promoter, and promotes H3K4 trimethylation, a histone mark of active transcription. Together, these results indicate that zebrafish *cul4a* regulates primitive erythropoiesis by promoting *scl-α and gata1* transcription.

SCL was originally identified as a proto-oncogene through the study of T-cell acute lymphoblastic lecukemia patients with a chromosomal translocation, and encodes a basic helix-loop-helix transcription factor^8,9,40^. The importance of SCL in the initiation of primitive and definitive hematopoiesis, erythrocyte and megakaryocyte differentiation, angiogenesis, and astrocyte development has been demonstrated in cell-based ex vivo assays as well as in knockout mouse models^41–44^. To our surprise, while knockdown of *cul4a* led to significant downregulation of *gata1*, one of the downstream genes of *scl* and *lmo2*, the markers for primitive myelopoiesis as well as definitive hematopoiesis were not found to be reduced, which is not due to the redundant role of Cul4b, since neither *cul4b*^*−/−*^ nor double knockout mutants exhibited a reduction in the expression of these markers. Previous study has elucidated that zebrafish produces two *scl* isoforms, *scl-α* and *scl-β*, which exert distinct functions in the regulation of primitive erythroid differentiation and definitive hematopoietic stem cell specification^39,45^. We thus examined the effect of *cul4a* knockdown on the expression of two *scl* isoforms, and revealed that knockdown of *cul4a* downregulated *scl-α*, but not *scl-β*. Further studies are required to elucidate how Cul4a complex regulates the temporal and spatial patterns of expression of the different isoforms. On the other hand, it has been identified that Lmo2 works together with Scl to regulate both primitive and definitive hematopoiesis^46^. The decreased *scl* levels led by *cul4a* depletion could attenuate the effect of downregulated *lmo2* on hematopoiesis. Intriguingly, Cul4a also directly regulates the expression of *gata1*, supporting that Cul4a plays more important roles in regulating *gata1* expression than that in regulating *scl* expression.

Epigenetic modifications have essential roles in cellular development and differentiation. For example, a recent study revealed that zebrafish TET2 plays an essential role in hematopoiesis by activating lineage-specific genes, *scl, gata1*, and *c-myb*, via DNA oxidative demethylation^47^. CUL4B complex was found to function as a transcriptional repressor by catalyzing histone H2A monoubiquitination at lysine 119, which facilitates the recruitment of repressive complexes PRC2, HDAC, and DNMT to chromatin and in turn catalyzes trimethylation of histone H3K27, histone deacetylation, and DNA methylation, respectively^48–50^. In contrast, CUL4A complex has been found to function as transcriptional activator. For example, Cul4A was reported to activate *ZEB1* in tumor progression and *tbx5* expression in zebrafish development by promoting H3K4 methylation^29,51^. In this study, we showed that Cul4a could bind to *scl-α* and *gata1* promoters, and promote H3K4 trimethylation. Future studies need to determine how CUL4A complex functions as a transcriptional activator for *scl-α* and *gata* expression. *Cul4a* knockout mice do not show obvious developmental or health defects except for male infertile^28^. *Cul4a* zebrafish morphants, on the other hand, failed to undergo heart looping^29^ and primitive erythropoiesis. While loss of function mutation of human *CUL4B* leads to an array of developmental and behavioral abnormalities^20,21^, and constitutive deletion of *Cul4b* in mouse is incompatible with embryonic development^22,23^, *cul4b* zebrafish morphants exhibit no remarkable phenotypes^29^. These results suggest that CUL4A and CUL4B may each have species-specific functions.

In summary, this study unveiled a function of Cul4a in primitive erythropoiesis in zebrafish. Cul4a binds to *scl* and *gata1* promoters and promotes their transcription during primitive erythropoiesis. Our study provides new insight into erythropoiesis, which may have general implications in regeneration medicine of anemia and related diseases.

## Materials and methods

### Zebrafish maintenance

The Tübingen strain and *Tg* transgene zebrafish were maintained in a circulating water system with a 14-h light/10-h dark cycle at 28.5 °C. The *Tg* transgene zebrafish was kindly provided by Professor Yiyue Zhang (South China University of Technology). Embryos were collected from adult fish after natural mating and raised at 28.5 °C in N-Phenylthiourea (PTU, Sigma Aldrich, St. Louis, MO, USA, P7629)/Holtfreter solution (0.003%, final) to prevent pigmentation implemented with 280 μg/L methylene blue (Sigma Aldrich, M4159). Four nanograms of Morpholinos (Gene Tools LLC, Philomath, OR, USA) was injected at the 1- to 2-cell stage of embryos with splice-blocking (MO: 5′-CTTGGGTCTGTCTGTAACACACAGA-3′), translation-blocking (MO1: 5′-GCTGGTGCTGAACATCTTCTGCCAT-3′), or control MO (CoMO: 5′-CTAGCGTCTCTCTCTAACACACACA-3′) that was used as the negative control as described previously^29^. For the rescue experiments, 80–100 pg mRNA was injected.

### Generation of cul4-depleted zebrafish embryos by CRISPR/Cas9 system

Six site-specific CRISPR-Cas9 sgRNAs for the zebrafish *cul4a* or *cul4b* gene were designed using the online software CRISPR design tool (http://www.crisprscan.org) CRISPRscan, and the primer sequences are listed in Table S1. We chose four highly efficient sgRNAs for *cul4a* and *cul4b*, respectively. To produce sgRNA templates for in vitro transcription, *cul4a* and *cul4b* sgRNAs were produced by in vitro transcription using the MEGAshortscript T7 Transcription Kit (Ambion Life Technologies, CA, USA) as described previously^31^. Zebrafish embryos were injected with 1 nL mixed solution containing 150 pg of sgRNA, 120 pg of cas9 protein (Script, Z03389-50, New-Jersey, USA), and 1.5 ng of phenol red (Sigma-Aldrich, P0290) tracer. After 24 hpf, 10 of the injected embryos were assayed for sgRNA activity by DNA extraction, PCR amplification, restriction digestion, and DNA sequencing.

The screening primers were designed around the *cul4a* (Exon15, 13, 11, 5, and 2) or *cul4b* (Exon1, 12, and 17) sgRNA target sites. The primer sequences were listed in Table S2. The fragments were cloned using the TA Cloning Kit (TAKARA, 6028, Japan) and then were sequenced.

### Whole-mount in situ hybridization (WISH)

WISH was performed using single-stranded RNA probes labeled with digoxigenin-uridine 5-triphosphate (Roche, Mannheim, Germany, 11277073910) essentially as described with minor changes^32,33^. Briefly, embryos were manually dechorionated, fixed in 4% PFA at 4 °C overnight. Embryos over 24 hpf were permeabilized using 10 μg/ml Proteinase K (Ambion, Austin, TX, USA, AM2546) in PBST (1 x PBS + 0.1% Tween-20) for 5 min and post-fixed in 4% PFA for 20 min at the room temperature. Hybridization was performed using 100 ng Digoxigenin-labeled *cul4a*, *scl*, *lmo2*, *gata1*, *hbbe3*, *pu.1*, *mpo*, and *runx1,* and *c-myb* probes synthetized using the T7 RNA polymerase (Promega, Fitchburg, WI, USA, P2075). The probes were detected by incubating the embryos with an anti-Digoxigenin antibody coupled to alkaline phosphase (Roche, 11093274910) diluted 1:2500 in blocking solution (Roche, 11921673001). The staining reaction was performed using NBT/BCIP (Sigma, N6639, B-8503) in staining buffer (100 mM Tris-HCl pH 9.5, 50 mM MgCl_2_, 100 mM NaCl, 0.1% Tween 20). All in situ probes were synthesized as described previously^34^. Plasmid clones for the generation of probes of zebrafish *c-myb* and *runx1*, were kindly provided by Professor Yiyue Zhang (South China University of Technology) and Professor Weijun Pan (Shanghai Institutes for Biological Sciences, Chinese Academy of Sciences). Plasmid clones of zebrafish *gata1* and *pu.1* were provided by Professor Yiyue Zhang. Plasmid clones of zebrafish *mpo* were provided by Travis Maslow in Harvard university. Other plasmid clones for generating probes of zebrafish *lmo2*, *hbbe3*, and full-lengh *scl* were kindly provided by Professor Anming Meng (Institute of Zoology, Chinese Academy of Sciences). Images were acquired using the Olympus SZX16 system (Olympus, Tokyo, Japan), and analyzed with Image-pro plus software.

### Quantitative real-time PCR(qRT-PCR)

Total mRNA was extracted with TRIzol (Ambion, 175803) from 24 hpf embryos (at least 100 embryos). RNA was used as template for cDNAs synthesis, which was performed using the RevertAid Reverse transcriptase (ThermoFisher, Rockford, IL, USA, EP0441). qRT-PCR was performed on a LightCycler 480 using SYBR Green (Roche, 04887352001). The sequence-specific primers were designed using Primer 5 software and shown in supplementary Table S3.

### *o*-dianisidine Staining

For detection of hemoglobin in red blood cells of zebrafish embryos, the staining was performed as described previously^35^.

### Whole-embryo quantitative chromatin immunoprecipitation(E-qChIP)

E-qChIP was performed as previously described with modifications^36^. Briefly, 24 hpf zebrafish embryos (at least 1000 per sample) were hand-dechorionated and fixed in 4% formaldehyde (final concentration) for 20 min at room temperature, followed by glycine (0.125 M) treatment for 5 min. The embryos were then homogenized in cell lysis buffer (10 mM Tris-HCl pH 7.5, 10 mM NaCl, 0.5% NP-40, and protease inhibitors), and incubated for 10 min on ice. Nuclei were collected by centrifugation, resuspended in nuclei lysis buffer (50 mM Tris-HCl pH 7.5, 10 mM EDTA, 1% SDS, and protease inhibitors), and incubated for 10 min. Chromatin was sonicated by Diagenode Bioruptor to yield fragments of 300–500 bp. Chromatin was pre-cleared with protein G agarose beads (Invitrogen, Carlsbad, CA, USA, 00507406), then incubated with antibodies with rotation overnight at 4 °C. Antibodies used were Cul4a (Abcam, ab72548), and H3K4me3 (Chemicon/Millpore, Billerica, MA, USA, 07-473). Beads were washed five times with RIPA wash buffer (50 mM HEPES pH 7.6, 1 mM EDTA, 0.7% Deoxycholic acid sodium, 1% NP-40, and 0.5% LiCl) and once with 1X Triton wash buffer (50 mM HEPES pH 7.6, 150 mM NaCl, and 1% Triton X-100) at 4 °C. Bound complexes were eluted from the beads at 65 °C with vortexing in elution buffer (1 M NaHCO_3_, 1% SDS). Cross-links were reversed for 6–12 h at 65 °C and the chromatin purified the DNA Clean-up Kit (CWBIO, Beijing, China, CW2301M). The primers used in qPCR were shown in supplementary Table S4.

### Statistical analysis

All experiments were repeated at least three times. Quantitative data are expressed as mean ± SD. Statistical significance was determined by the Student’s *t-*test. *P* value of less than 0.05 was considered statistically significant.

## Supplementary information

Supplemental figure 1

supplemental Table S1

supplemental Table S2

supplemental Table S3

supplemental Table S4
