# Supplementary material for: Cul4a promotes zebrafish primitive erythropoiesis via upregulating scl and gata1 expression
Source: Cell Death Dis. 2019 May 17;10(6):388. doi: 10.1038/s41419-019-1629-7 (PMC6525236; doi:10.1038/s41419-019-1629-7)
Supplement: Supplementary file 1 — Supplemental figure 1 [file 41419_2019_1629_MOESM1_ESM.docx]

**
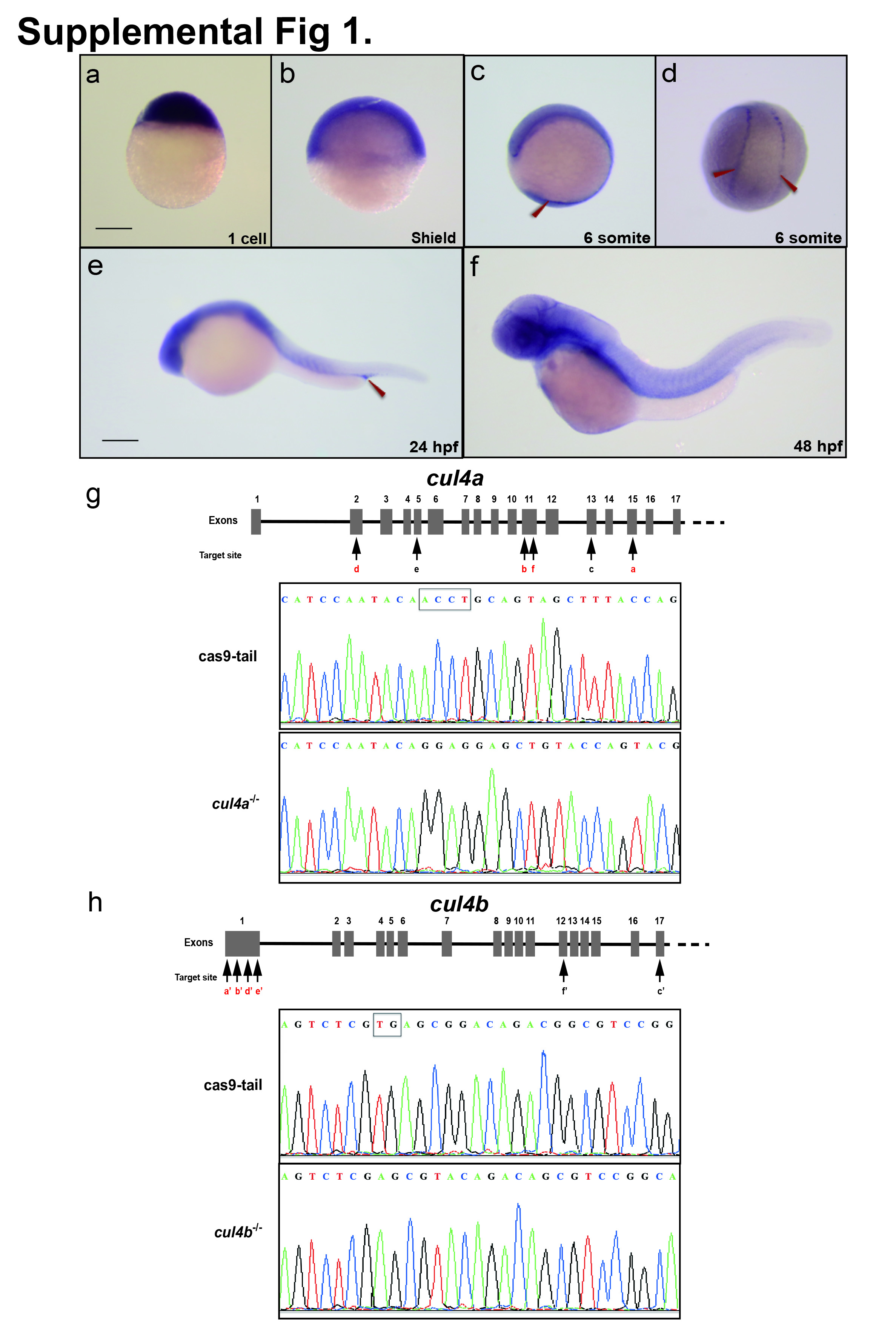
**

**Fig. S1** **a-f** Expression of *cul4a* was analyzed via WISH at 1 cell (**a**), 6 hpf (**b**), 12 hpf (**c** and **d**), 24 hpf (**e**) and 48 hpf (**f**) stages with the *cul4a* antisense probe. Red arrowheads indicate PLPM and ICM regions*.* Lateral views are shown with anterior to the left (**c, e, f**), and dorsal views are shown with anterior to the top (**d**). **g** Diagram of the target site in the zebrafish *cul4a* and *cul4b* genome. **h** Representative sequences from control and *cul4a* or *cul4b* mutants. All scale bars represent 250 μm.
