# Supplementary material for: Cul4a promotes zebrafish primitive erythropoiesis via upregulating scl and gata1 expression
Source: Cell Death Dis. 2019 May 17;10(6):388. doi: 10.1038/s41419-019-1629-7 (PMC6525236; doi:10.1038/s41419-019-1629-7)
Supplement: Supplementary file 2 — supplemental Table S1 [file 41419_2019_1629_MOESM2_ESM.docx]

**Table S1. The sequences of sgRNAs used in CRISPR/Cas9 system**

**SgRNA Scaffold Primer (tail)** AAAAGCACCGACTCGGTGCCACTTTTTCAAGTTGATAACGGACTAGCCTTATTTTAACTTGCTATTTCTAGCTCTAAAAC

*cul4a*-sgRNA -a TAATACGACTCACTATAGGAGGCAGATGGACGTCCATGTTTTAGAGCTAGAA

*cul4a*-sgRNA -b TAATACGACTCACTATAGGACGGTGTGTTCTCCGGAGGTTTTAGAGCTAGAA

*cul4a*-sgRNA -c TAATACGACTCACTATAGGTGGGCAAGAGCGCTTCAGGTTTTAGAGCTAGAA

*cul4a*-sgRNA -d TAATACGACTCACTATAGGGTAGGAGTCTGTCAGCTTTGTTTTAGAGCTAGAA

*cul4a*-sgRNA -e TAATACGACTCACTATAGGCTCCGTGTATGGATAAGGTGTTTTAGAGCTAGAA

*cul4a*-sgRNA -f TAATACGACTCACTATAGGGATTCAAAGCTCCGTGCTTGTTTTAGAGCTAGAA

*cul4b*-sgRNA –a’ TAATACGACTCACTATAGGGAGCGGACAGACGGCGTCGTTTTAGAGCTAGAA

*cul4b*-sgRNA –b’ TAATACGACTCACTATAGGGGCTCTTGGGCTGGCGGTGTTTTAGAGCTAGAA

*cul4b*-sgRNA –c’ ATTTAGGTGACACTATAGAGGACATTAAGCTGGCCACGTTTTAGAGCTAGAA

*cul4b*-sgRNA –d’ ATTTAGGTGACACTATAGGCTCCGTGTATGGATAAGGGTTTTAGAGCTAGAA

*cul4b*-sgRNA –e’ ATTTAGGTGACACTATAGGCTCCGTGTATGGATAAGGGTTTTAGAGCTAGAA

*cul4b*-sgRNA –f’ ATTTAGGTGACACTATAGGGATTCAAAGCTCCGTGCTGTTTTAGAGCTAGAA
