# Supplementary material for: Cul4a promotes zebrafish primitive erythropoiesis via upregulating scl and gata1 expression
Source: Cell Death Dis. 2019 May 17;10(6):388. doi: 10.1038/s41419-019-1629-7 (PMC6525236; doi:10.1038/s41419-019-1629-7)
Supplement: Supplementary file 3 — supplemental Table S2 [file 41419_2019_1629_MOESM3_ESM.docx]

**Table S2. The sequences of primer pairs used in screening CRISPR-targeted mutations**

| **Gene Symbol** | **Forward(5’-3’)** | **Reverse(5’-3’)** |
| --- | --- | --- |
| cul4a-ex15 | 5’ CATCATGCTCACGCTCATCCTT | 5’ CTCAGCAGGCAGATGGACGTC |
| cul4a-ex11 | 5’ GTACACACACACAGACTTGTT | 5’ TGAGGAGAAACAGCGCACATG |
| cul4a-ex13 | 5’ AGTGTGTGTCTACATTTATGCG | 5’ CAGGTCGCTTCCTGTTCACAGG |
| cul4a-ex2 | 5’ TACATTGGTTTATAGATGGACA | 5’ TCCTGTGAACAGGAAGCGACC |
| cul4a-ex5 | 5’ AGAGTCTCTGGACAGCCTGTCG | 5’ GCACACACACACACACGTTATG |
| cul4b-ex1 | 5’ GCAGCTTGTAATCCAACGGA | 5’ GTTGTGAACATCAGAAGCTGC |
| cul4b-ex17 | 5’ GTGATATGTGGGTAATGCACT | 5’ GTGTGTCACGATTTTTCATAC |
| cul4b-ex12 | 5’ GTATGTACCTGCTGTCTG | 5’ AGTCTATGAAGCTTAGCTG |
