# Supplementary material for: Cul4a promotes zebrafish primitive erythropoiesis via upregulating scl and gata1 expression
Source: Cell Death Dis. 2019 May 17;10(6):388. doi: 10.1038/s41419-019-1629-7 (PMC6525236; doi:10.1038/s41419-019-1629-7)
Supplement: Supplementary file 4 — supplemental Table S3 [file 41419_2019_1629_MOESM4_ESM.docx]

**Table S3. The sequences of primer pairs used in Quantitative real-time PCR**

| **Gene Symbol** | **Forward(5’-3’)** | **Reverse(5’-3’)** |
| --- | --- | --- |
| *gata1* | 5’ TCTGAGCCTTCTCGTTGGGTGTC | 5’ TCCTGGAGCCTGGGACTGTCTT |
| *hbbe3* | 5’ CTTTCCAGGACTTTGTTCGTTT | 5’ ACCGTGGTTCCGTGCTTC |
| *cmyb* | 5’ AGTTACTTCCGGGAAGAACCG | 5’ AGAGCAAGTGGAAATGGCACC |
| *runx1* | 5’ GGGACGCCAAATACGAACCT | 5’ GCAGGACGGAGCAGAGGAA |
| *mpo* | 5’ TGATGTTTGGTTAGGAGG | 5’ GAGCTGTTTTCTGTTTTGGTG |
| *cul4a* | 5’ TAGCGTTGTTGTTATCGTCATTAT | 5’ TGTCAGCTTGGGTCTGTCTTTA |
| *scl* | *5’ GGGAAACAGGAAGGCACGAC* | *5’ CGCTTGACTCGATTGCTAGGAT* |
| *lmo2* | *5’* GATGCTTGGAATCTGGCGTACA | 5’ CCATCTGCCGCACAAAACG |
| *gapdh* | *5’ CCAACTGCCTGGCTCCTT* | *5’ CCCATCAACGGTCTTCTGTG* |
| *scl-α* | *5’*GAAAGACTCAATTAGTTTGA | 5’ GGGATTCAGCAGCCCTATC |
| *scl-β*  *pu.1* | *5’ GGTGCAGACCACCGAGCTG*  *5’*CGGATCCATCCCAGCAGTCG | 5’ TCGTTGATTTCAACCTCATA  5’CTCTAGACCGTCTTTCCGTAG |
