# Supplementary material for: Cul4a promotes zebrafish primitive erythropoiesis via upregulating scl and gata1 expression
Source: Cell Death Dis. 2019 May 17;10(6):388. doi: 10.1038/s41419-019-1629-7 (PMC6525236; doi:10.1038/s41419-019-1629-7)
Supplement: Supplementary file 5 — supplemental Table S4 [file 41419_2019_1629_MOESM5_ESM.docx]

**Table S4. The sequences of primer pairs used in E-qChIP.**

| **Gene Symbol** | **Forward(5’-3’)** | **Reverse(5’-3’)** |
| --- | --- | --- |
| Ch-scl-1 | 5’ ATTATCAAGTGTCTGTCTTCTCCTC | 5’ ATGTCAAAAAGAAAATACAATG |
| Ch-scl-2 | 5’ GACATGTTTAATTTCTAAAGCTTCC | 5’ TAAAATGTAATAACTGGTGCAT |
| Ch-scl-3 | 5’ ATGCACCAGTTATTACATTTTATGA | 5’ TATTCACTTTATTAATTTGGGGT |
| Ch-scl-4 | 5’ CAACCCCAAATTAATAAAGTGAATA | 5’ CAGTGTTGTAGTTTTTATTGCAG |
| Ch-scl-5 | 5’ ATCTGCAATAAAAACTACAACACTG | 5’ GTTATTAAGGTAAGCAGACAAA |
| Ch-scl-6  Ch-gata1-1  Ch-gata1-2  Ch-gata1-3  Ch-gata1-4  Ch-gata1-5  Ch-gata1-6  Ch-gata1-7 | 5’ ACCTTAATAACCTGCCTAACATTTT  5’ GCCTGATAAACTGAATTTAGTA  5’ GAACTGCATGTCTTTGCAGG  5’ GCATCGAGTGTGTAGCGAC  5’ CTGATTATTCAGCAAAATGT  5’ CTGATTATTCAGCAAAATGT  5’ CAAAGACTGAAGGAGATAAGC  5’ CCTCTCCACTCTCACACCTCC | 5’ AAAATAACTCAGCAGATTGAGG  5’ GTCGTGCAAAAGAATTATGTGAC  5’ ACCAAAATTCAGAGTAGTCG  5’ TTATCGATTGAAGTATGCGG  5’ GGTTATGTAAAACTAAAGCT  5’ AACTTAAAATTTAAGGTTGG  5’ GTTCAAATGAATAGTGGAGG  5’ CAGTCTCAGAGCTGGAGTAG |
